# Public Preference Heterogeneity and Predicted Uptake Rate of Upper Gastrointestinal Cancer Screening Programs in Rural China: Discrete Choice Experiments and Latent Class Analysis

**DOI:** 10.2196/42898

**Published:** 2023-07-10

**Authors:** Ruyue Liu, Qiuxia Li, Yifan Li, Wenjian Wei, Siqi Ma, Jialin Wang, Nan Zhang

**Affiliations:** 1 Center for Health Management and Policy Research, School of Public Health, Cheeloo College of Medicine Shandong University Jinan China; 2 National Health Commission Key Lab of Health Economics and Policy Research Shandong University Jinan China; 3 School of Public Health Weifang Medical University Weifang China; 4 Shandong Cancer Hospital and Institute, Shandong First Medical University and Shandong Academy of Medical Sciences Jinan China

**Keywords:** upper gastrointestinal cancer, screening programs, discrete choice experiment, latent class logit model, public preference heterogeneity, uptake rate

## Abstract

**Background:**

Rapid increases in the morbidity and mortality of patients with upper gastrointestinal cancer (UGC) in high-incidence countries in Asia have raised public health concerns. Screening can effectively reduce the incidence and mortality of patients with UGC, but the low population uptake rate seriously affects the screening effect.

**Objective:**

We aimed to determine the characteristics that influence residents’ preference heterogeneity for a UGC-screening program and the extent to which these characteristics predict residents’ uptake rates.

**Methods:**

A discrete choice experiment was conducted in 1000 residents aged 40-69 years who were randomly selected from 3 counties (Feicheng, Linqu, and Dongchangfu) in Shandong Province, China. Each respondent was repeatedly asked to choose from 9 discrete choice questions of 2 hypothetical screening programs comprising 5 attributes: screening interval, screening technique, regular follow-up for precancerous lesions, mortality reduction, and out-of-pocket costs. The latent class logit model was used to estimate residents’ preference heterogeneity for each attribute level, their willingness to pay, and the expected uptake rates.

**Results:**

Of the 1000 residents invited, 926 (92.6%) were included in the final analyses. The mean age was 57.32 (SD 7.22) years. The best model contained 4 classes of respondents (Akaike information criterion=7140.989, Bayesian information criterion=7485.373) defined by different preferences for the 5 attributes. In the 4-class model, out of 926 residents, 88 (9.5%) were assigned to class 1, named as the negative latent type; 216 (3.3%) were assigned to class 2, named as the positive integrated type; 434 (46.9%) were assigned to class 3, named as the positive comfortable type; and 188 (20.3%) were assigned to class 4, named as the neutral quality type. For these 4 latent classes, “out-of-pocket cost” is the most preferred attribute in negative latent type and positive integrated type residents (45.04% vs 66.04% importance weights), whereas “screening technique” is the most preferred factor in positive comfortable type residents (62.56% importance weight) and “screening interval” is the most valued attribute in neutral quality type residents (47.05% importance weight). Besides, residents in different classes had common preference for painless endoscopy, and their willingness to pay were CNY ¥385.369 (US $59.747), CNY ¥93.44 (US $14.486), CNY ¥1946.48 (US $301.810), and CNY ¥3566.60 (US $552.961), respectively. Residents’ participation rate could increase by more than 89% (except for the 60.98% in class 2) if the optimal UGC screening option with free, follow-up for precancerous lesions, 45% mortality reduction, screening every year, and painless endoscopy was implemented.

**Conclusions:**

Public preference heterogeneity for UGC screening does exist. Most residents have a positive attitude toward UGC screening, but their preferences vary in selected attributes and levels, except for painless endoscopy. Policy makers should consider these heterogeneities to formulate UGC-screening programs that incorporate the public’s needs and preferences to improve participation rates.

## Introduction

Upper gastrointestinal cancer (UGC; including gastric cancer and esophageal cancer) is one of the most common malignant neoplasms worldwide, and 1.60 million new UGC cases were reported and 1.31 million UGC deaths occurred in 2020, with China alone accounting for more than half of the cases and deaths, respectively [1]. Among patients diagnosed with UGC, the 5-year survival rate is approximately 30% [2]. The main reason for the poor prognosis is that most patients are diagnosed at an advanced stage when tumors are not resectable and nonsurgical therapeutic modalities are ineffective [3]. Nevertheless, UGC can be detected and treated with endoscopic screening at an earlier stage, with a survival rate of >90% [4]. Endoscopic screening, the gold standard for the early diagnosis of UGC, has been widely adopted in many countries and proven to be highly effective in reducing the morbidity and mortality rates of UGC [5,6]. Furthermore, recent studies have confirmed that endoscopic screening is a more cost-effective screening method for UGC than no screening [7-9]. However, the effectiveness of endoscopic screening is reduced by the low population uptake rate.

In some high-income countries, such as Japan and South Korea, the participation rate is lower than 50% despite the launch of nationwide endoscopic screening programs for gastric cancer for a long time [10,11]. Similarly, as a low-income country with high UGC incidence and mortality, China has performed endoscopic screening for patients with gastric cancer and esophageal cancer in more than 110 high-risk areas throughout the country since 2005, but residents’ compliance to date was still only 48.62% [6]. This low uptake rate for UGC screening has become a huge public health challenge and needs to be addressed to maximize the benefits that can be achieved by endoscopic screening. Recent studies have shown that the characteristics (attributes and levels) of the UGC-screening program may impact whether individuals participate in the screening [12-16]. Nevertheless, if and how these characteristics affect residents’ screening preferences remains poorly understood.

The discrete choice experiment (DCE) is a stated preference method that has been used from 1990s to obtain individuals’ preferences for health care and for a wide range of health care topics [17]. In a DCE, participants are presented with alternative options that are systematically described according to several attributes and are asked to make a choice among these options [18]. Their choice can be analyzed using a discrete choice model. The latent class logit (LCL) model is the most commonly used choice model for exploring individuals’ preference heterogeneity, which permits the potential classification of the target population and estimation of their preference heterogeneity in different classes [19]. However, to date, no published studies have used LCL to explore individuals’ UGC-screening preference heterogeneity.

Therefore, in this study, we constructed a DCE and used the LCL model to elicit public preference heterogeneity regarding UGC-screening programs and explore the influence of personal characteristics on the variation, willingness to pay (WTP), and uptake rate in their choices. These results will be helpful for policy makers in understanding the heterogeneity of public preferences, resulting in a sustainable and effective UGC-screening policy.

## Methods

### Sampling and Participants

This is an extension of our previously published study [12], which also used contemporaneous data but where the new methods are used to obtain more essential and meaningful findings and conclusions. In our study, a stratified (cluster) random sampling method was used to select participants. First, located in the east, central, and west of Shandong Province, 3 cities (Weifang, Taian, and Liaocheng) were chosen as sample areas, which represent high, medium, and low economic levels according to the gross domestic product (GDP) per capita (2020), respectively. Then, 3 counties (Linqu, Feicheng, and Dongchangfu) were selected from each sample area as the survey points, respectively. Finally, rural residents aged 40 to 69 years in 2 to 4 villages were randomly chosen from each county for a face-to-face DCE questionnaire survey.

### DCE Design

#### Overview

In this study, a DCE was conducted to determine the factors (attributes and attribute levels) that influence residents’ preference heterogeneity to participate in a UGC-screening program and the weightage that residents give to the selected attributes and attribute levels. The DCE was developed according to the methodological standards issued by the International Society for Pharmacoeconomics and Outcomes Research (ISPOR) [20]. The design process is as follows. More details on the DCE design process, sample size calculation, and data collection are available in our previously published study [12].

#### Selection of Attributes and Attribute Levels

The attributes and attribute levels of the DCE were derived from a literature review [5,12-14,21-29], expert interviews, and focus group discussions with residents of the target population.

Finally, the survey results described 5 attributes with 2 to 4 levels: out-of-pocket costs (CNY ¥0 [US $0], CNY ¥100 [US $15.503], CNY ¥300 [US $46.511], and CNY ¥500 [US $77.519]), screening interval (every year, every 2 years, every 5 years, and once in a lifetime), screening technique (endoscopy and painless endoscopy), mortality reduction (15%, 30%, 45%, and 60%), and regular follow-up for precancerous lesions (yes or no). Notably, the “mortality reduction” attribute in this study refers to the respondents’ expected reduction extent of mortality because of UGC if they chose to participate in UGC screening.

#### Questionnaire Design

SAS (version 9.4) software was used to design the questionnaire. The combination of 5 attributes with 2 to 4 levels yielded 256 (ie, 4^3^ × 2^2^) possible scenarios using full factor design [30]. To minimize the respondents’ burden, a smaller fractional factorial design was selected according to the D-efficiency criteria. The final design randomly generated 16 choice sets into 2 blocks with 8 choice tasks. For each choice task, 2 screening programs (options A and B) and an opt-out option (whether to choose to be screened in real life) were included. In addition, a rationality test (choice set 1=choice set 5) was included in the DCE to investigate residents’ understanding of the questionnaire [31]. Table 1 presents an example of a discrete choice task.

The questionnaire also contained sociodemographic questions, including age, sex (male and female), educational level, and medical insurance. As a result, the questionnaire had 2 sections: the first section comprised sociodemographic questions and the second comprised information about the 9 DCE choice sets (Multimedia Appendices 1-4).

**Table 1:** Example of a discrete choice task^a^.

Attributes	Option A	Option B
Out-of-pocket costs (CNY ¥ or US $)^b^	100 or 15.503	300 or 46.511
Screening interval	Every 2 years	Every year
Regular follow-up for precancerous lesions	Yes	No
Mortality reduction (%)	15	45
Screening technique	Endoscopy	Painless endoscopy
Which of these options would you prefer?	Option A	Option B
Would you screen as you choose in real life?	Yes	No

^a^See the study by Liu et al [12] for more details.

^b^CNY ¥6.45=US $1.00.

#### Pilot Study

Before the main study, we conducted a pilot test with residents of the target population to ascertain whether respondents could manage the length of the questionnaire and examine the validity, acceptability, and intelligibility of the questionnaire.

In total, 30 residents were recruited for the pilot test and interviewed by 8 trained investigators. Respondents were provided with descriptions of the attributes and levels and were given the opportunity to comment on the questionnaire design and layout. They found the questionnaire acceptable and easy to complete. No major changes were made to the questionnaires.

### Sample Size and Data Collection

Calculating the optimal DCE sample size is complicated by the fact that it depends on the question format, complexity of the choice tasks, degree of heterogeneity in the target population, and need to conduct subgroup analyses [21,32]. However, there is a generally accepted rule of thumb proposed by Orem and Johnson [33,34] for calculating sample size:



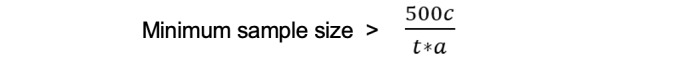



The required sample size depends on the largest number of levels for any 1 attribute (*c*), number of alternatives per choice task (*a*), and number of choice tasks (*t*). Therefore, this questionnaire required at least 125 respondents [(500 × 4) / (8 × 2)] to estimate the main effect alone. Because 2 blocks were included in the design, there was a need for at least 300 respondents (125 × 2). Other studies found that the sample size of foreign DCE studies in the field of health care ranged from 150 to 1200, and 77% of the studies had a sample size 600, whereas most of the studies in China had a sample size below 600 [30,35,36]. Therefore, we combined the abovementioned literature reviews, the need for LCL model analysis, and an expected response rate of 20% to determine a total sample size of 1000 residents.

The final survey was conducted from April 1, 2021, to May 31, 2021. A total of 1000 permanent residents of the sample area were invited to participate in the survey, and they were assisted in completing the questionnaire by 8 trained investigators. A 2-step survey was conducted to interview the residents. In the first step, respondents were asked to describe their basic information, such as age, sex, and health insurance. In the second step, they were repeatedly asked to choose between 2 alternative options from the per-choice task (a total of 9 choice tasks).

### Statistical Analysis

#### Overview

Statistical analyses were performed using Stata 16.0 software, with the commands *lclogit2* and *lclogitml2* for the analysis of the preference data. All attributes other than cost were treated as classification variables coded by dummy variables, whereas the cost parameter was modeled as a continuous variable to allow for WTP estimation. Sociodemographic data were summarized using descriptive statistics.

#### LCL Model and Model Fitting

The LCL model is the most commonly used preference heterogeneity model, assuming that preferences differ across respondents and that respondents can be grouped probabilistically according to distinct preference classes, each corresponding to a unique program preference [37,38]. It is based on random utility theory [39]:

U = ASC + β_1_ (screening interval: every year) + β_2_ (screening interval: every 2 years) + β_3_ (screening interval: every 5 years) + β_4_ (follow-up: yes) + β_5_ (follow-up: yes) + β_6_ (mortality reduction: 30%) + β_7_ (mortality reduction: 45%) + β_8_ (mortality reduction: 60%) + β_9_ (screening technique: endoscopy screening) + β_10_ (screening technique: painless endoscopy screening) + β_11_ (out-of-pocket costs) + ε

where U refers to the utility of the respondents’ (in particular, latent class) choice of option across the different choice sets in the formal investigation stage; ASC (alternative specific constant) represents a fixed constant term (reflecting the screening attitudes of respondents in different classes), which was used to capture unobservable influences beyond attributes present in the choice sets; β provides quantitative information on the strength of preference for each attribute level; and ε is an unobservable random component. A positive β coefficient indicates that the attribute level combination is preferred relative to the reference level, whereas a negative β coefficient indicates the opposite. The higher the absolute value of β coefficient, the stronger is the preference for that level relative to the reference level. Then, using the expectation-maximization algorithm, we constructed an LCL model with different numbers of classes. The Akaike information criterion (AIC) and Bayesian information criterion (BIC) were used to compare the model fit, with lower AIC and BIC values indicating better models [40].

#### Parameter Estimation

Importance weights are a measure of the importance of an attribute relative to other attributes in the model for an individual’s preferences [41]. It is calculated by dividing the maximum utility of an attribute by the total utility of all the attributes.

WTP refers to the fee that residents of different classes were willing to spend or want to be compensated for the change in a certain attribute level in a specific program. To calculate the respondents’ WTP, the estimation of cost attributes was used as a measure of the marginal utility of money. The ratio of the value of the coefficient of other attribute levels to the negative of the cost attribute 
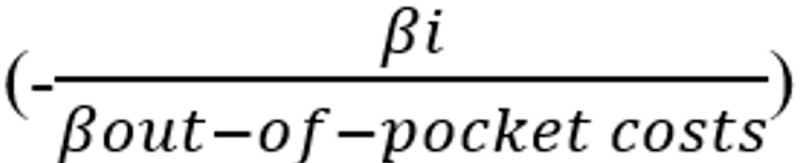
 was calculated to elicit respondents’ WTP for contribution to a UGC-screening program.

From a policy perspective, the uptake rate is a useful output for estimating the effect of policies yet to be implemented, such as the change in the participation rate of rural residents with increasing screening costs [39]. The logit probability of choosing alternative *i* rather than alternative *j* is given by the following equation where *x* is a vector of the attribute coefficients:



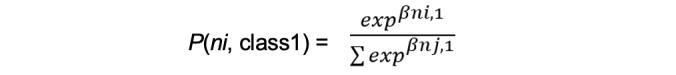



With the level of attribute improving from *k* (baseline reference level) to *g*, the change in the uptake rate when choosing the baseline program is given by the following equation:



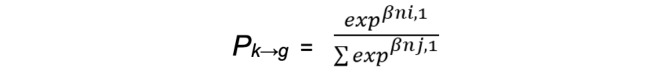



### Ethics Approval

The Institutional Ethical Review Board of Shandong Cancer Hospital and Institute approved this study (reference no. SDTHEC201909001).

## Results

### Study Participation

A total of 959 of 1000 invited residents completed the questionnaire (for a response rate of 95.9%), of which 33 residents failed the consistency test. The sensitivity analysis indicated that there were no substantial differences in preference between the full sample and those who passed the consistency test. However, from the model fitting results, the likelihood, AIC, and BIC values of model 2 (removing samples of residents who did not pass the consistency test) were smaller than those of model 1, indicating that model 2 has a better fit (Multimedia Appendix 5). Considering the accuracy of the results, the 926 respondents who passed the consistency test were finally included in the preference estimation using the LCL model. Table 2 presents the characteristics of the 926 respondents. In all, 44.7% (414/926) of respondents were aged 50 to 59 years, and 95.9% (888/926) had a partner. Most respondents (743/926, 80.2%) had no family history of cancer, and 56.2% (520/926) had experience in cancer screening.

**Table 2:** Characteristics of study participants^a^.

Characteristics			Respondents (n=926)
Age (years), mean (SD)			57.32 (7.22)
**Sex, n (%)**			
	Male	315 (34)	
	Female	611 (66)	
**Age group (years), n (%)**			
	40-49	139 (15)	
	50-59	414 (44.7)	
	60-70	373 (40.3)	
**Marital status^b^, n (%)**			
	With a partner	888 (95.9)	
	Without a partner	38 (4.1)	
**Annual family income (CNY ¥>)^c^, n (%)**			
	＜10,000	434 (46.9)	
	10,000-29,999	294 (31.7)	
	≥30,000	198 (21.4)	
**Location, n (%)**			
	Linqu	322 (34.8)	
	Feicheng	310 (33.5)	
	Dongchangfu	294 (31.7)	
**Family history of cancer^d^, n (%)**			
	Yes	183 (19.8)	
	No	743 (80.2)	
**Screening for cancer, n (%)**			
	Ever	520 (56.2)	
	Never	406 (43.8)	

^a^For more details, see the study by Liu et al [12].

^b^Marital status: with a partner, reflecting that the individual is married and the spouse is alive; and without a partner, including single, divorced, and widowed.

^c^The per capita gross domestic product in 2020 in Linqu, Feicheng, and Dongchangfu was CNY ¥39,910, CNY ¥80,696, and CNY ¥50,726, respectively. The average exchange rate between US $ and CNY ¥ in 2021 was US $1=CNY ¥6.45.

^d^History of cancer in blood relatives, including parents, grandparents, siblings, uncles, aunts, and cousins.

### Model Fitting Results

To select the appropriate number of classes, different numbers of classes were tested, from 2 to 5. The AIC and BIC values were used to select the final number of clusters that best fit the data. As presented in Table 3, there is a gradual decline in the values of AIC and BIC with an increase in the latent class number. When the number of latent classes is 4, the values reach a minimum (AIC=7140.989; BIC=7485.373) and subsequently increase. Accordingly, the 4-class model has the best fit for the data. The respondents’ preference heterogeneity was analyzed by constructing an LCL model with 4 classes.

**Table 3:** Results of model fitting under different classes.

Class	Observation, n	*df*	LL^a^	AIC^b^	BIC^c^
2	22,224	21	−4139.361	8320.722	8488.910
3	22,224	32	−3728.246	7520.492	7776.777
4	22,224	43	−3527.495	7140.989	7485.373
5	22,224	54	−3533.606	7175.213	7607.695

^a^LL: likelihood.

^b^AIC: Akaike information criterion.

^c^BIC: Bayesian information criterion.

### Preferences Estimation

Table 4 shows that respondents’ preferences varied at each attribute level, except for painless endoscopy. The detailed preference differences and the naming of different classes are as follows.

The residents (88/926, 9.5%) in class 1 responded negatively for UGC screening (β=3.372; *P*<.001), but they preferred lower screening costs. Theoretically, the respondents’ negative screening attitudes can be changed once the cost of screening programs is reduced, so they were defined as the negative latent type (NLT).

**Table 4:** Parameters estimation results of latent class logit model.

Attributes and levels			Class 1 (n=88; class share=0.095)				Class 2 (n=216; class share=0.233)			Class 3 (n=434; class share=0.469)				Class 4 (n=188; class share=0.203)		
			β coefficient^a^		*P* value^b^		β coefficient		*P* value	β coefficient		*P* value		β coefficient		*P* value
ASC^c^ (opt-out^d^)			3.372		*<.001*		−3.843		*<.001*	−2.165		*<.001*		.076		.85
**Screening interval (once in a lifetime^e^)**																
	Every year	.514		.19		.432		*.003*		−.280	.33		2.294		*<.001*	
	Every 2 years	.298		.37		.015		.92		.141	.72		2.336		*<.001*	
	Every 5 years	.764		*.03*		.359		*.02*		.286	.37		1.277		*<.001*	
**Follow-up (yes^e^)**																
	No	.396		*.01*		−.274		*<.001*		−.535	*.01*		−.247		*.02*	
**Morality reduction (15%^e^)**																
	30%	−.161		.60		−.039		.82		.605	.13		.503		.007	
	45%	−.477		.13		.366		*.03*		.147	.67		.867		*<.001*	
	60%	−.285		.37		−.208		*.02*		.124	.69		1.142		*<.001*	
**Screening technique (endoscopy^e^)**																
	Painless endoscopy	2.804		*<.001*		.612		*<.001*		4.173	*<.001*		1.088		*<.001*	
Out-of-pocket cost			−.007		*<.001*		−.007		*<.001*	−.002		.004		−.0003		.32

^a^β coefficient: it reflects the values of each attribute level and the horizontal regression coefficient.

^b^Italicized *P* values denote a significance level at *P*<.05.

^c^ASC: alternative specific constant.

^d^Specific constant item for opt-out.

^e^Reference, reflecting the reference level for each attribute.

The residents (216/926, 23.3%) in class 2 responded most positively for UGC screening (β=−3.843; *P*<.001), and they preferred the program with every year, follow-up, 45% mortality reduction, and painless endoscopy, indicating that they had equal consideration in each attribute, so they were defined as the positive integrated type (PIT).

The residents (434/926, 46.8%) in class 3 responded positively for UGC screening (β=−2.165; *P*<.001), and they preferred follow-up, painless endoscopy, and lower out-of-pocket cost attribute levels over the screening interval and mortality reduction attributes. This group of people paid more attention to the comfort and experience of the screening process; therefore, they were defined as a positive comfortable type (PCT).

The residents (188/926, 20.3%) in class 4 had a neutral attitude toward UGC screening (β=.076; *P*>.05), and they preferred screening programs with shorter screening intervals, painlessness, follow-up, and a much lower risk of death regardless of the costs, indicating that they place more value on the quality and effect of the screening program, so they were defined as the neutral quality type (NQT).

### Participants’ WTP

Table 5 shows that NLT residents were willing to spend CNY ¥385.369 (US $59.747) to improve screening technique, that is, to upgrade endoscopy to painless endoscopy; PIT residents had more WTP for screening every year (CNY ¥65.966 [US $10.227]) compared with once in a lifetime, and they should be compensated CNY ¥41.776 (US $6.477) if there is no regular follow-up for precancerous lesions; PCT residents had the highest WTP for painless endoscopy (CNY ¥1946.482 [US $301.780]), and the participants should be compensated CNY ¥249.753 (US $ 38.721) if there is no follow-up after screening in theory. For the NQT population, there was no statistically substantial difference in costs attribute.

**Table 5:** Results of participants’ willingness to pay (WTP).

Attributes and levels			Class 1 (n=88)				Class 2 (n=216)				Class 3 (n=434)					Class 4 (n=188)	
			WTP	*P* value^a^		WTP			*P* value	WTP			*P* value		WTP		*P* value
**Screening interval (once in a lifetime^b^)**																	
	Every year	70.622		.19	65.97			*<.001* ^b^		−130.71		.35		7524.14			.33
	Every 2 years	40.950		.39	2.35			.91		65.56		.74		7660.38			.34
	Every 5 years	104.981		*.051*	54.73			*.02*		133.22		.33		4187.49			.33
**Follow-up (yes^b^)**																	
	No	54.381		.09	−41.78			*<.001*		−249.75		*.02*		−810.56			.38
**Morality reduction (15%^b^)**																	
	30%	−22.088		.06	−5.98			.81		281.99		.18		1649.85			.37
	45%	−65.495		.14	55.93			.02		68.79		.67		2841.79			.36
	60%	−39.177		.38	−31.78			.13		57.63		.71		3744.69			.35
**Screening technique (endoscopy^b^)**																	
	Painless endoscopy	385.369		*<.001*	93.44			*<.001*		1946.48		*<.001*		3566.60			.34

^a^Italicized *P* values denote a significance level at *P*<.05.

^b^Reference, reflecting the reference level in each attribute; WTP reflects residents’ willingness to pay for a certain screening program.

### Personal Characteristics Influencing Factors

Sociodemographic characteristics influencing factors are presented in Table 6. Class 4 was automatically identified as the reference class in the LCL model. Accordingly, the residents living in Linqu were included in class 1 easily, whereas male respondents and those with an experience of UGC screening were more likely to be excluded from class 1. Residents who had a family history of cancer were more likely to be assigned to class 2 than those without a family history. Compared with female respondents, male respondents were less likely to be included in class 3, and residents aged 60 to 69 years were more likely to be included in class 3.

**Table 6:** Personal characteristics influencing factors.

Attributes and levels		Class 1		Class 2		Class 3	
		β (SE)	*P* value^a^	β (SE)	*P* value	β (SE)	*P* value
**Location (Feicheng^b^)**							
	Linqu	2.083 (0.386)	*<.001*	−.008 (0.325)	.98	.883 (0.269)	*<.001*
	Dongchangfu	−.575 (0.518)	.27	.181 (0.319)	.57	.530 (0.279)	.06
**Annual family income^c^ (¥<10,000^b^)**							
	10,000-29,999	−.143 (0.427)	.74	−.392 (0.342)	.25	0.067 (0.291)	.81
	≥30,000	.371 (0.383)	.33	.409 (0.318)	.19	0.428 (0.283)	.13
**Sex (female^b^)**							
	Male	−1.095 (0.356)	*.002*	−.094 (0.271)	.73	−.608 (0.230)	.008
**Age group (years; 40-49^b^)**							
	60-69	1.244 (0.492)	*.01*	.951 (0.406)	*.01*	.844 (0.315)	.007
	50-59	0.592 (0.445)	.18	.640 (0.361)	.07	.338 (0.270)	.21
**Education level (university and above^b^)**							
	Below junior high school	−2.341 (1.003)	*.02*	.327 (1.266)	.79	−.496 (0.791)	.53
	Junior high and high school	−2.737 (0.957)	.004	.124 (1.237)	.92	−.802 (0.762)	.29
**Family history of cancer^d^(no^b^)**							
	Yes	−.647 (0.363)	.08	−.769 (0.303)	.01	−.396 (0.238)	.09
**Screening for cancer^e^ (never^b^)**							
	Ever	−.657 (0.310)	.03	−.217 (0.272)	.42	−.144 (0.223)	.52
	Constant	.801 (1.114)	.47	−.535 (1.348)	.69	.722 (0.869)	.41

^a^Italicized *P* values denote a significance level at *P*<.05.

^b^Reference, reflecting the reference level.

^c^The average exchange rate between US $ and CNY ¥ in 2021 was US $1=CNY ¥6.45.

^d^Family history of cancer, including parents, grandparents, siblings, uncles, aunts, and cousins having a history of cancer.

^e^Screening for cancer means attending endoscopy screening at any time in the past.

### Relative Importance of Attributes

**Figure 1:**
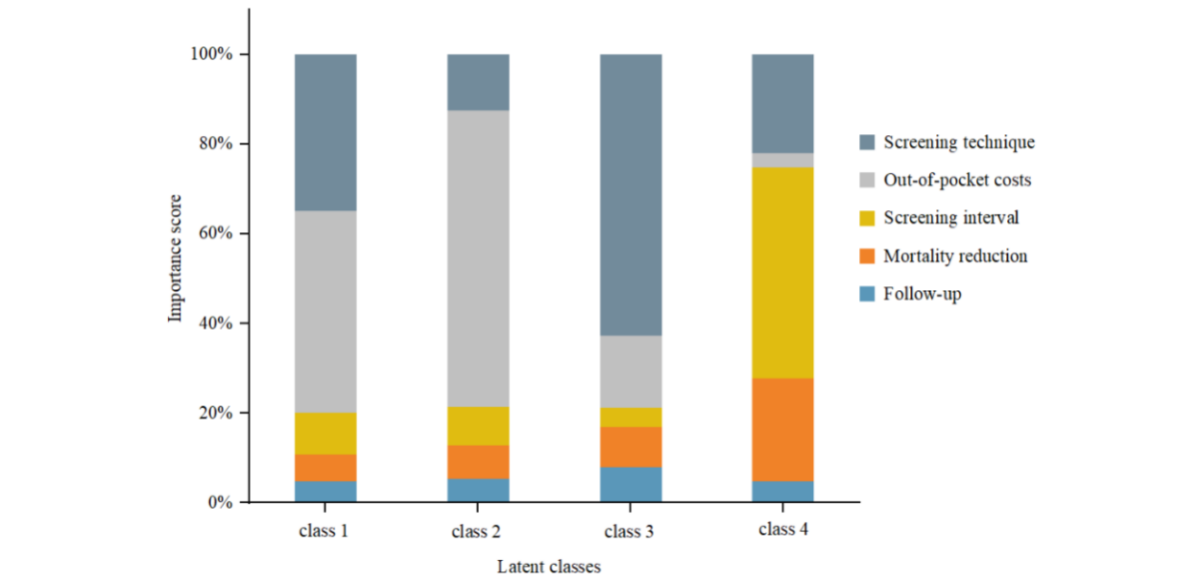
Relative importance of attributes in different classes. Relative scores expressed in percent as weighted out of 100.

As presented in Figure 1, we calculated the importance weights for the 5 attributes in different classes. The out-of-pocket cost attribute was the factor that most influenced class 1 and class 2 residents’ willingness to participate in UGC screening (45.04% and 66.04% importance weights, respectively). The screening technique was the most important for class 3 respondents (62.56% importance weight). The screening interval was the most important for class 4 residents (47.05% importance weight) and the out-of-pocket cost was not important (3.07% importance weight).

### Uptake Rate

**Figure 2:**
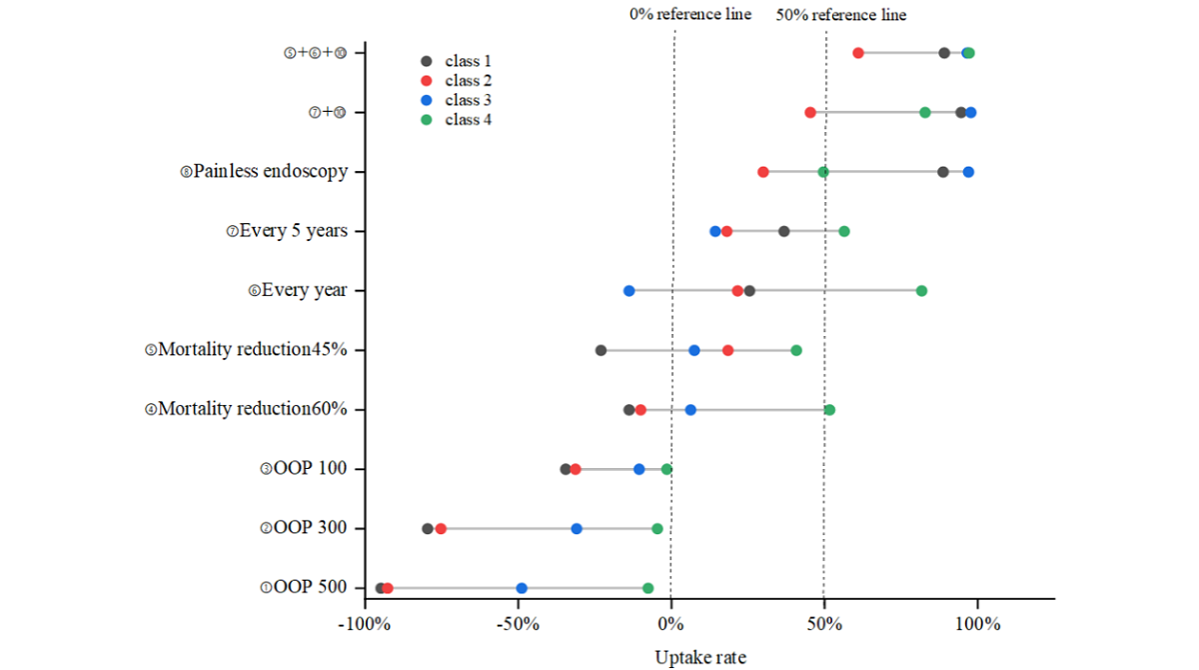
Uptake rate of residents in different classes. Null percent and 50% reference line represents the reference in uptake rate as 0% and 50%, respectively. The horizontal comparison between points can see the change of the participation rate of residents in different classes under the same attribute level, and the longitudinal comparison between points can show the change of participation rate of individuals in the same class under the change of different attribute levels. OOP: out-of-pocket costs.

Figure 2 depicts the change in choice probability for different classes of respondents, as attributes and levels were changed at the baseline level (baseline: once in a lifetime, follow-up, 15% mortality reduction, endoscopy, and CNY ¥0). The screening uptake rate of residents in classes 1, 2, and 3 dramatically decreased by ≥48% (94.84%, 92.67%, and 48.91%, respectively) when the out-of-pocket costs increased to CNY ¥500 (US $71.903), whereas the uptake rate in class 4 remained constant. For class 1 and class 3 residents, the participation rate was strongly driven by painless endoscopy, with an increase of 88.66% and 96.97%, respectively. Among residents in class 4, the participation rate would increase by 81.69% if the screening interval was improved from once in a lifetime to every year. The expected uptake rate for the best UGC-screening program (CNY ¥0, 45% mortality reduction, every year screening, and painless endoscopy) would increase by 89% and above in classes 1, 3, and 4, and class 2 residents’ participation rate would remain ≥60%.

## Discussion

### Principal Findings

To the best of our knowledge, this is the first study to explore public preference heterogeneity for a UGC-screening program by classifying the population. This study is a continuation and extension of our previous study [12]. We used novel analytic methods to explore population preference heterogeneity and found that preference heterogeneity exists when residents choose to participate in UGC-screening programs. Preference heterogeneity was explorable: the target population was divided into 4 categories, including NLT, PIT, PCT, and NQT. Residents in different classes had different preferences for the selected attributes and attribute levels, except for the screening technique attribute (preferably painless endoscopy). On the basis of this, we found that the optimal screening strategy with free, 45% mortality reduction, screening every year, and painless endoscopy could ensure that the screening participation rate of class 1, class 3, and class 4 residents reached ≥89%, and the screening uptake rate of residents in class 2 could reach 60.98%.

Nearly half (434/926, 46.8%) of the respondents were divided into the PCT group, indicating that they paid more attention to their experience and comfort levels regarding the screening procedure rather than the outcome. This is consistent with previous findings, in which the author found that residents’ screening participation rate was significantly associated with severe pain and endoscopy experience [13]. Similarly, a DCE study of immediate family members with UGC indicated that participants preferred a pain-none program, increasing the participation rate by 58.85% compared with the reference level (pain-mild) [42]. Further analysis revealed that the uptake rate of residents of different classes was mostly driven by painless endoscopy. Accordingly, if painless endoscopic screening technology can be widely promoted, it will be able to satisfy the screening needs and preferences of residents in different categories, greatly improving population screening enthusiasm and participation rate. Areas with a good economic level and sufficient screening resources should take painless endoscopy as the essential element, paying more attention to the population screening experience.

Compared with other classes, NQT residents valued the screening quality and outcome most, such as screening interval and mortality reduction attributes (47.05% and 23% importance weights, respectively), and their screening compliance increased once the accuracy of endoscopy was improved. An unlabeled DCE assessing the attributes of an optimal esophageal adenocarcinoma screening test found that test accuracy generally outweighs the importance of potential pain and discomfort [13]. Consistent with our results, various DCEs evaluating preferences for breast, cervical, and colorectal cancer screening reported that attributes related to test accuracy were more important than attributes related to the screening procedure [43-45]. Although these studies targeted different cancer types and populations, their results provide face validity for the results of this study. This study provides an important reference for the clinical development of scientific and effective screening. Improving the accuracy of screening techniques and health care services is the main way to satisfy residents’ growing demand for health care services and to increase their participation rate in a UGC screening program.

All residents, except those in class 4, strongly preferred the UGC-screening program with lower costs, particularly for the residents in class 1, and their negative attitude will be theoretically improved if the screening fee is reduced. These results are consistent with a recent South Australian study in which the uptake rate of participants decreased by 48% after the screening fee was changed from A$0 to A$500 (US $0 to US $328.62) [24]. Li et al [42] also pointed out that the probability of screening program selection increased by 11.30% when the cost changed from CNY ¥600 (US $86.28) to CNY ¥200 (US $28.76). Other studies regarding lung cancer screening and colorectal cancer screening found that cost-related attributes had an important impact on the residents’ participation rate, and they all preferred the screening program with a lower cost [18,46]. However, it is unrealistic to provide a nationwide free UGC-screening strategy because of the large population size and limited medical resources. These results provide support and guidance for policy makers to explore the multifinancing and cost sharing of different interventions.

Only 9.5% of the respondents had a negative response for UGC screening, which is consistent with the screening status in rural China. These residents did not proactively seek medical attention or predisease screening influenced by their family income and the traditional concept of medical treatment. Other studies have also pointed out that some residents do not think it is necessary to screen before the onset of noticeable symptoms [47]. Therefore, policy makers should strengthen primary prevention and emphasize the importance of screening for cancer to promote more high-risk groups to take the initiative to screen, reduce their morbidity and mortality, and reduce the socioeconomic burden.

Differences in health care delivery systems across countries may affect public-related medical needs and preferences. A label DCE study conducted in the Netherlands indicated that respondents preferred video capsule over saliva swab and least preferred endoscopy, but a study performed in China showed that respondents have a strong preference for endoscopy [12,22]. In addition, we found that the population in different countries has a common preference for out-of-pocket cost attributes, preferring free or less-expensive screening programs [13,42,48,49]. Notably, in this study, we did not investigate the preference differences between health care systems in China and other countries. Accordingly, caution should be exercised when applying our results to other countries with different health care systems.

### Limitations

This study has some limitations that should be considered. First, DCE as a stated preference method differs from revealed preferences (ie, a difference between what people say they will choose and what they actually choose) [50]. The revealed preferences should be examined after implementing the UGC-screening programs. Second, the development of UGC-screening programs depends not only on the population uptake rate but also on the cost-effectiveness of those programs. In this study, we mainly analyzed the residents’ screening uptake rate. However, other studies have reported that UGC screening is always more cost-effective than not screening [7,8]. Third, the respondents were selected from Shandong Province and interviewed during a specific period; thus, the results need to be carefully applied to all rural residents in China and other resource-limited countries.

### Conclusions

Most rural residents have a positive attitude toward UGC screening, but they expressed preference heterogeneity in selected attributes and levels, except for painless endoscopy. The optimal UGC-screening program that is free with 45% mortality reduction, screening every year, and painless endoscopy should be implemented to maximize participation rates if resources permit. In areas with limited health resources, the population uptake rate can be controlled by adjusting steerable screening attributes such as screening intervals and out-of-pocket costs. In addition, differentiated screening programs suitable for residents belonging to different categories should be developed within certain regions. These findings will provide input for the design of a UGC-screening strategy that incorporates the public’s preference heterogeneity to improve their participation rate and provide practical evidence for other resource-limited countries with high UGC incidence and mortality.

## Appendix

Multimedia Appendix 1 Discrete choice experiment (DCE) questionnaires (block A) in Chinese.

Multimedia Appendix 2 Discrete choice experiment (DCE) questionnaires (block A) in English.

Multimedia Appendix 3 Discrete choice experiment (DCE) questionnaires (block B) in Chinese.

Multimedia Appendix 4 Discrete choice experiment (DCE) questionnaires (block B) in English.

Multimedia Appendix 5 Sensitivity analysis.

## Abbreviations

AIC: Akaike information criterion
ASC: alternative specific constant
BIC: Bayesian information criterion
DCE: discrete choice experiment
GDP: gross domestic product
ISPOR: International Society for Pharmacoeconomics and Outcomes Research
LCL: latent class logit
NLT: negative latent type
NQT: neutral quality type
PCT: positive comfortable type
PIT: positive integrated type
UGC: upper gastrointestinal cancer
WTP: willingness to pay

## Data Availability

The data sets generated and analyzed during this study are available from the corresponding author upon reasonable request.
