# Supplementary material for: Public Preference Heterogeneity and Predicted Uptake Rate of Upper Gastrointestinal Cancer Screening Programs in Rural China: Discrete Choice Experiments and Latent Class Analysis
Source: JMIR Public Health Surveill. 2023 Jul 10;9:e42898. doi: 10.2196/42898 (PMC10366669; doi:10.2196/42898)
Supplement: Multimedia Appendix 3 [file publichealth_v9i1e42898_app3.docx]

编号：|__|__|__||__|__|__|

**居民癌症防治需求调查问卷(block B)**

调查对象住址： 市 区（县） 街道（乡/镇） 社区（村）

调 查 日 期： 年 月 日

**知情同意书**

项目介绍

尊敬的受访者您好，我们是山东第一医科大学的研究生，为全面了解居民癌症防治需求，分析居民癌症筛查、诊治的偏好，为改善全省癌症防治服务提供依据，现组织开展癌症防治需求调研。调研内容主要包括基本信息和癌症筛查偏好，您提供的信息将为相关政策的制定提供重要参考。

信息收集及使用

您选择参与，将意味着您将接受问卷调查。调查大约会占用您20~30分钟时间，研究过程中收集到的所有信息都将被编码，您的姓名和个人信息不会出现在数据当中，将被永久保密。您的姓名也不会出现在本项目的任何公开发行物中。

签字

对于以上信息我已经清楚地阅读并理解，而且我所有的问题都得到了满意的回答，并对调查员的说明感到满意。我明白：我的参与是自愿的，而且我可以随时中断我的参与。签署这份知情同意书并不意味着放弃我的任何法律权利，我将参与此次研究。

感谢您参与本次调查，您提供的信息将为我们的项目提供重要的参考，此次调查**不记姓名**，不做个人评价，我们将对**调查信息严格保密**。**答案及选项均无对错之分，请放心填写您的实际情况，不要留空项。**

调 查 对 象 签 名 ________

代 签 人 签 名 _________

与调查对象的关系 _________

签名日期： 年 月 日

第一部分：基本信息

- - 1. 您的性别： 1=男 2=女
    2. 您的出生日期是： 年 月
    3. 民族： 1=汉族 2=其他
    4. 文化程度：_________

1=没上过学 2=小学 3=初中

4=高中/中专 5=大专 6=本科及以上， .

- - 1. 婚姻状况： 1=未婚 2=已婚 3=离婚 4=丧偶
    2. 您的职业：_________

1=公务员 /事业单位人员2=企业人员/工人 3=在校学生

4=个体户 5=自由职业者 6=农民/农民工

7=无业人员 8=退休，退休前职业 9=其他， .

- - 1. 您去年一年的家庭总收入为（包括所有收入来源，如工资、养老金、抚养费、福利、投资、利息、出租房屋、庄稼收成等） 元

7.1您自我感觉您的家庭经济状况处于什么水平：_________

1= 差 2= 一般 3= 良好 4= 优越

- - 1. 您参加的医疗保险有（可多选）： _________

1=城镇职工医疗保险 2=城乡居民医疗保险； 3=商业医疗保险；

4=公费医疗； 5=无； 6=其他， .

- - 1. 您目前的身体状况是：_________

1=健康 2=患有慢性病，具体是： . 3=伤残

- - 1. 您的血缘亲属（父母、祖父母、外祖父母、兄弟姐妹、叔姑舅姨、堂兄弟姐妹、表兄弟姐妹）中是否有人曾患癌症： . 1=是 2=否
    2. 您既往是否出现过一些上消化道症状（如腹胀、腹痛、恶心嗳气、反酸、烧心、吐血、黑便等）： 1=有，具体是： . 2=无
    3. 您是否参加过癌症筛查： 1=是 2=否

12.1如果有，是哪种形式：_________

1=单位组织的筛查 2=政府组织的筛查项目

3=个人自费筛查 4=其他， .

第二部分：上消化道癌内镜筛查偏好

***上消化道癌（包括食管癌、胃癌等）**是我国的高发癌症，**内镜筛查**是发现早期上消化道癌和癌前病变的重要手段。通过筛查和及时干预能阻断癌症的发生、显著提高治愈率和降低医疗费用。

****内镜筛查**是一种侵入性操作，检查前需禁食、禁水6小时以上，分为普通胃镜和无痛胃镜，普通胃镜有一定不适性（如恶心、肿胀、异物感等）。无痛胃镜需静脉注射麻醉剂，检查过程中不会存在明显的不适，且准确性较高。

**属性解释：**

1. 自付费用，指在财政补助或医保报销后，个人每次参与内镜筛查需自付的费用；
2. 筛查间隔，指重复进行内镜筛查的时间间隔；
3. 癌前病变定期随访，指是否对筛查出的癌前病变，如重度萎缩性胃炎、低级别上皮内瘤变等进行定期随访、复查；
4. 上消化道癌死亡风险降低程度，指与不参加内镜筛查者相比，个体因参与内镜筛查而降低的上消化道癌死亡风险；
5. 筛查技术，分为普通胃镜和无痛（麻醉）胃镜。

请根据列出的相关信息，在下列9套筛查方案中选择您更倾向的内镜筛查方式（在相应选择项下的“□”中打“√”），并进一步选择您在现实生活中，是否会按照所选方案参加筛查。

**方案1**

| 属性 | 选项A | 选项B |
| --- | --- | --- |
| 自付费用 | 0元 | 500元 |
| 筛查间隔 | 2年1次 | 终生1次 |
| 癌前病变定期随访 | 无 | 有 |
| 上消化道癌死亡风险降低程度 | 15% | 45% |
| 筛查技术 | 无痛（麻醉）胃镜 | 普通胃镜 |
| **您的选择是？** | □ | □ |
| **现实生活中，您会按照上述选择参加筛查吗？** | 会 | 不会 |

**方案2**

| 属性 | 选项A | 选项B |
| --- | --- | --- |
| 自付费用 | 500元 | 100元 |
| 筛查间隔 | 2年1次 | 5年1次 |
| 癌前病变定期随访 | 无 | 有 |
| 上消化道癌死亡风险降低程度 | 15% | 30% |
| 筛查技术 | 普通胃镜 | 无痛（麻醉）胃镜 |
| **您的选择是？** | □ | □ |
| **现实生活中，您会按照上述选择参加筛查吗？** | 会 | 不会 |

**方案3**

| 属性 | 选项A | 选项B |
| --- | --- | --- |
| 自付费用 | 300元 | 500元 |
| 筛查间隔 | 1年1次 | 终生1次 |
| 癌前病变定期随访 | 有 | 无 |
| 上消化道癌死亡风险降低程度 | 30% | 60% |
| 筛查技术 | 普通胃镜 | 无痛（麻醉）胃镜 |
| **您的选择是？** | □ | □ |
| **现实生活中，您会按照上述选择参加筛查吗？** | 会 | 不会 |

**方案4**

| 属性 | 选项A | 选项B |
| --- | --- | --- |
| 自付费用 | 100元 | 500元 |
| 筛查间隔 | 1年1次 | 5年1次 |
| 癌前病变定期随访 | 无 | 有 |
| 上消化道癌死亡风险降低程度 | 45% | 30% |
| 筛查技术 | 普通胃镜 | 无痛（麻醉）胃镜 |
| **您的选择是？** | □ | □ |
| **现实生活中，您会按照上述选择参加筛查吗？** | 会 | 不会 |

**方案5**

| 属性 | 选项A | 选项B |
| --- | --- | --- |
| 自付费用 | 0元 | 500元 |
| 筛查间隔 | 2年1次 | 终生1次 |
| 癌前病变定期随访 | 有 | 无 |
| 上消化道癌死亡风险降低程度 | 60% | 15% |
| 筛查技术 | 无痛（麻醉）胃镜 | 普通胃镜 |
| **您的选择是？** | □ | □ |
| **现实生活中，您会按照上述选择参加筛查吗？** | 会 | 不会 |

**方案6**

| 属性 | 选项A | 选项B |
| --- | --- | --- |
| 自付费用 | 0元 | 300元 |
| 筛查间隔 | 终生1次 | 5年1次 |
| 癌前病变定期随访 | 有 | 无 |
| 上消化道癌死亡风险降低程度 | 30% | 60% |
| 筛查技术 | 普通胃镜 | 无痛（麻醉）胃镜 |
| **您的选择是？** | □ | □ |
| **现实生活中，您会按照上述选择参加筛查吗？** | 会 | 不会 |

**方案7**

| 属性 | 选项A | 选项B |
| --- | --- | --- |
| 自付费用 | 0元 | 100元 |
| 筛查间隔 | 终生1次 | 2年1次 |
| 癌前病变定期随访 | 有 | 无 |
| 上消化道癌死亡风险降低程度 | 15% | 30% |
| 筛查技术 | 无痛（麻醉）胃镜 | 普通胃镜 |
| **您的选择是？** | □ | □ |
| **现实生活中，您会按照上述选择参加筛查吗？** | 会 | 不会 |

**方案8**

| 属性 | 选项A | 选项B |
| --- | --- | --- |
| 自付费用 | 100元 | 300元 |
| 筛查间隔 | 5年1次 | 1年1次 |
| 癌前病变定期随访 | 无 | 有 |
| 上消化道癌死亡风险降低程度 | 45% | 60% |
| 筛查技术 | 无痛（麻醉）胃镜 | 普通胃镜 |
| **您的选择是？** | □ | □ |
| **现实生活中，您会按照上述选择参加筛查吗？** | 会 | 不会 |

**方案9**

| 属性 | 选项A | 选项B |
| --- | --- | --- |
| 自付费用 | 100元 | 300元 |
| 筛查间隔 | 1年1次 | 2年1次 |
| 癌前病变定期随访 | 有 | 无 |
| 上消化道癌死亡风险降低程度 | 60% | 45% |
| 筛查技术 | 无痛（麻醉）胃镜 | 普通胃镜 |
| **您的选择是？** | □ | □ |
| **现实生活中，您会按照上述选择参加筛查吗？** | 会 | 不会 |
