# Supplementary material for: Public Preference Heterogeneity and Predicted Uptake Rate of Upper Gastrointestinal Cancer Screening Programs in Rural China: Discrete Choice Experiments and Latent Class Analysis
Source: JMIR Public Health Surveill. 2023 Jul 10;9:e42898. doi: 10.2196/42898 (PMC10366669; doi:10.2196/42898)
Supplement: Multimedia Appendix 4 [file publichealth_v9i1e42898_app4.docx]

Number：|__|__|__||__|__|__|

**Survey questionnaire on residents' cancer prevention and treatment needs (block B)**

Address： City District (County) Street Village

Survey date： year month day

**Informed Consent Form**

Introduction

Hello, we are postgraduates of Shandong First Medical University. In order to comprehensively understand the residents’ demand for cancer prevention and treatment, analyze the preference for cancer screening, and provide data support for improving cancer prevention and treatment services, we are organizing a survey. The survey mainly includes basic information, residents’ preference of upper gastrointestinal cancer screening. The results will provide important reference for policers to formulate the relevant policies.

Information Collection and Use

The survey will take approximately 20-30 minutes of your time, and all information collected during the study will be coded so that your name and personal information will not appear in the data and will be kept permanently confidential. Your name will also not appear in any public distribution of this project.

Signature

I have clearly read and understood the above information, and I am satisfied with the investigator's instructions. I understand that: my participation is voluntary and that I may discontinue my participation at any time. Signing this informed consent form does not waive any of my legal rights to participate in this study.

Thank you for participating in this survey, the information will provide important reference for our project, the survey does not record names, do not do personal evaluation, we will be strictly confidential survey information. Answers and options are not right or wrong, please feel free to fill in your actual situation, do not leave empty items.

Signatures of participants___________

Signed by the undersigned ___________

Relationship with participants__________

date： year month day

Part I：Basic Information

- - 1. Your gender: 1=Man 2=Women
    2. Your birth date: year month
    3. Nationalities: 1=Chinese 2=Other
    4. Education level: _________

1= Never attended school 2= Primary School 3= Middle School

4= High School/Junior College 5= College 6= Bachelor degree or above, .

- - 1. Marital status： 1=Not married 2= Married 3= Divorce 4= Widowhood
    2. Your profession：_________

1= Civil Servants / Career Staff 2= Enterprise personnel/workers 3= Students

4= Self-employed 5= Freelancer 6= Farmers / Migrant Workers

7= Unemployed people 8= Retirement, pre-retirement occupation____ 9=Others, .

- - 1. Your total household income for the past year (including all sources of income, such as wages, pensions, alimony, benefits, investments, interest, rental housing, crop harvests, etc.) RMB

7.1 Where do you feel your family's financial situation is at? _________

1= Poor 2= Fair 3= Good 4= Excellent

- - 1. You are covered by the medical insurance (multiple choices possible): _____

1=Medical insurance for urban workers 2=Medical insurance for urban and rural residents 3=Commercial medical insurance 4=Publicly funded medical insurance

5=None 6=Other, _________

- - 1. Your current physical condition is: _________

1=Healthy 2=Having a chronic disease, specifically: _____. 3=Disabled

- - 1. Whether any of your blood relatives (parents, grandparents, siblings, uncles, aunts, cousins, cousins) ever had cancer? 1=Have 2=Not have
    2. Have you had any previous upper gastrointestinal symptoms (e.g., bloating, abdominal pain, nausea and belching, acid reflux, heartburn, vomiting blood, black stools, etc.). 1=Have，specifically: . 2=Not have
    3. Have you ever attended a cancer screening: 1=Have 2=Not have

12.1 If so, which form: _________

1=Unit-organized screening 2=Government-organized screening program

3=Individual self-pay screening 4=Other, _________

Part 2：Upper Gastrointestinal Cancer Endoscopy Screening Preferences

***** **Upper gastrointestinal cancer (UGC, including esophageal cancer and stomach cancer)** is a highly prevalent cancer in China. Endoscopic screening is an important tool for detecting early-stage UGC and precancerous lesions. Screening and timely intervention can interrupt the occurrence of cancer, significantly improve cure rates and reduce medical costs.

****Endoscopic screening** Endoscopic screening is an invasive operation that requires fasting and water fasting for more than 6 hours before the examination, and is divided into general endoscopy and painless endoscopy, which has some discomfort (such as nausea, swelling, foreign body sensation, etc.). Painless endoscopy requires intravenous anesthetic, and there is no significant discomfort during the examination, and the accuracy is higher.

**Attribute explanation：**

1. Out-of-pocket costs refers to the costs for each individual to participate in endoscopic screening after financial assistance or health insurance reimbursement.
2. Screening interval means that the time interval between repeated endoscopic screenings.
3. Regular follow-up for precancerous lesions, meaning whether the precancerous lesions detected by screening, such as severe atrophic gastritis and low-grade intraepithelial neoplasia, are regularly followed up and reviewed.
4. Mortality reduction means the reduction in the risk of death from upper gastrointestinal cancer as a result of an individual's participation in endoscopic screening compared to those who do not participate in endoscopic screening.
5. Screening technique，which was divided into endoscopy and painless endoscopy in this study.

Based on the information listed, please select your preferred endoscopic screening option from the nine screening programs listed below (tick the appropriate option) and further select whether you would participate in screening according to your chosen option in real life.

**Program 1**

| Attributes | Option A | Option B |
| --- | --- | --- |
| Out-of-pocket costs | ¥0 | ¥500 |
| Screening interval | Every 2 years | Once in a lifetime |
| Regular follow-up for precancerous lesions | No | Yes |
| Mortality reduction | 15% | 45% |
| Screening technique | Painless endoscopy | Endoscopy |
| **Which of these options would you prefer?** | □ | □ |
| **Would you choose** **to be screened in a real life?** | Yes | No |

**Program 2**

| Attributes | Option A | Option B |
| --- | --- | --- |
| Out-of-pocket costs | ¥500 | ¥100 |
| Screening interval | Every 2 years | Every 5 year |
| Regular follow-up for precancerous lesions | No | Yes |
| Mortality reduction | 15% | 30% |
| Screening technique | Endoscopy | Painless endoscopy |
| **Which of these options would you prefer?** | □ | □ |
| **Would you choose to be screened in a real life?** | Yes | No |

**Program 3**

| Attributes | Option A | Option B |
| --- | --- | --- |
| Out-of-pocket costs | ¥300 | ¥500 |
| Screening interval | Every year | Once in a lifetime |
| Regular follow-up for precancerous lesions | Yes | No |
| Mortality reduction | 30% | 60% |
| Screening technique | Endoscopy | Painless endoscopy |
| **Which of these options would you prefer?** | □ | □ |
| **Would you choose to be screened in a real life?** | Yes | No |

**Program 4**

| Attributes | Option A | Option B |
| --- | --- | --- |
| Out-of-pocket costs | ¥100 | ¥500 |
| Screening interval | Every year | Every 5 years |
| Regular follow-up for precancerous lesions | No | Yes |
| Mortality reduction | 45% | 30% |
| Screening technique | Endoscopy | Painless endoscopy |
| **Which of these options would you prefer?** | **□** | **□** |
| **Would you choose to be screened in a real life?** | **Yes** | **No** |

**Program 5**

| Attributes | Option A | Option B |
| --- | --- | --- |
| Out-of-pocket costs | ¥0 | ¥500 |
| Screening interval | Every 2 years | Once in a lifetime |
| Regular follow-up for precancerous lesions | Yes | No |
| Mortality reduction | 60% | 15% |
| Screening technique | Painless endoscopy | Endoscopy |
| **Which of these options would you prefer?** | □ | □ |
| **Would you choose to be screened in a real life?** | Yes | No |

**Program 6**

| Attributes | Option A | Option B |
| --- | --- | --- |
| Out-of-pocket costs | ¥0 | ¥300 |
| Screening interval | Once in a lifetime | Every 5 years |
| Regular follow-up for precancerous lesions | Yes | No |
| Mortality reduction | 30% | 60% |
| Screening technique | Endoscopy | Painless endoscopy |
| **Which of these options would you prefer?** | □ | □ |
| **Would you choose to be screened in a real life?** | Yes | No |

**Program 7**

| Attributes | Option A | Option B |
| --- | --- | --- |
| Out-of-pocket costs | ¥0 | ¥100 |
| Screening interval | Once in a lifetime | Every 2 years |
| Regular follow-up for precancerous lesions | Yes | No |
| Mortality reduction | 15% | 30% |
| Screening technique | Painless endoscopy | Endoscopy |
| **Which of these options would you prefer?** | □ | □ |
| **Would you choose to be screened in a real life?** | Yes | No |

**Program 8**

| Attributes | Option A | Option B |
| --- | --- | --- |
| Out-of-pocket costs | ¥100 | ¥300 |
| Screening interval | Every 5 years | Every year |
| Regular follow-up for precancerous lesions | No | Yes |
| Mortality reduction | 45% | 60% |
| Screening technique | Painless endoscopy | Endoscopy |
| **Which of these options would you prefer?** | □ | □ |
| **Would you choose to be screened in a real life?** | Yes | No |

**Program 9**

| Attributes | Option A | Option B |
| --- | --- | --- |
| Out-of-pocket costs | ¥100 | ¥300 |
| Screening interval | Every year | Every 2 years |
| Regular follow-up for precancerous lesions | Yes | No |
| Mortality reduction | 60% | 45% |
| Screening technique | Painless endoscopy | Endoscopy |
| **Which of these options would you prefer?** | □ | □ |
| **Would you choose to be screened in a real life?** | Yes | No |
