# Supplementary material for: Public Preference Heterogeneity and Predicted Uptake Rate of Upper Gastrointestinal Cancer Screening Programs in Rural China: Discrete Choice Experiments and Latent Class Analysis
Source: JMIR Public Health Surveill. 2023 Jul 10;9:e42898. doi: 10.2196/42898 (PMC10366669; doi:10.2196/42898)
Supplement: Multimedia Appendix 5 [file publichealth_v9i1e42898_app5.docx]

**Supplementary Table 1.** Sensitivity analysis

| **Attributes and levels** | **Model Ⅰ** | | | | **Model Ⅱ** | | | | |
| --- | --- | --- | --- | --- | --- | --- | --- | --- | --- |
|  | ***β* coefficient (SE)** | ***P* value** | **95%CI** | | ***β* coefficient (SE)** | | ***P* value** | **95%CI** | |
| ASC (Opt-out) | -6.829 (0.719) | 0.000 | -8.238 | -5.419 | | -7.004 (0.676) | 0.000 | -8.329 | -5.680 |
| Screening interval | | | | | | | | | |
| Once in a lifetime (Ref) | | | | | | | | | |
| Every year | 1.184 (0.087) | 0.000 | 1.013 | 1.355 | | 1.138 (0.086) | 0.000 | 0.969 | 1.307 |
| Every 2 years | 1.122 (0.097) | 0.000 | 0.933 | 1.312 | | 1.065 (0.096) | 0.000 | 0.877 | 1.252 |
| Every 5 years | 0.971 (0.105) | 0.000 | 0.764 | 1.177 | | 0.957 (0.104) | 0.000 | 0.753 | 1.161 |
| Regular follow-up for precancerous lesions | | | | | | | | | |
| Yes (Ref) |  |  |  |  | |  |  |  |  |
| No | -0.243 (0.050) | 0.000 | -0.342 | -0.144 | | -0.221 (0.050) | 0.000 | -0.319 | -0.122 |
| Mortality reduction | | | | | | | | | |
| 15% (Ref) |  |  |  |  | |  |  |  |  |
| 30% | 0.068 (0.089) | 0.445 | -0.107 | 0.243 | | 0.070 (0.088) | 0.423 | -0.102 | 0.242 |
| 45% | 0.225 (0.103) | 0.030 | 0.022 | 0.427 | | 0.209 (0.101) | 0.040 | 0.010 | 0.408 |
| 60% | 0.191 (0.082) | 0.020 | 0.030 | 0.352 | | 0.201(0.081) | 0.013 | 0.042 | 0.359 |
| Screening technique | | | | | | | | | |
| Endoscopy (Ref) | | | | | | | | | |
| Painless (anesthesia) endoscopy | 2.927 (0.148) | 0.000 | 2.638 | 3.217 | | 2.880 (0.151) | 0.000 | 2.584 | 3.177 |
| Out-of-pocket costs | -0.004 (0.000) | 0.000 | -0.005 | -0.004 | | -0.004 (0.000) | 0.000 | -0.005 | -0.004 |
| Sample size | 926 | | | | | 959 | | | |
| Observation | 22,224 | | | | | 23016 | | | |
| LL | -3604.771 | | | | | -3800.666 | | | |
| AIC | 7249.541 | | | | | 7641.331 | | | |
| BIC | 7409.720 | | | | | 7802.210 | | | |

Note: * P <0.05, * * P <0.01; ASC (Opt-out)：a specific constant item for opt-out; Ref: reference, which reflects a reference level in each attribute; β coefficient: which reflects the values of each attribute level and the horizontal regression coefficient; SE, standard error; 95% CI, 95% confidence interval
